# Supplementary figures and images for: ITRAQ-Based Proteomic Analysis of Wheat (Triticum aestivum) Spikes in Response to Tilletia controversa Kühn and Tilletia foetida Kühn Infection, Causal Organisms of Dwarf Bunt and Common Bunt of Wheat
Source: Biology (Basel). 2022 Jun 5;11(6):865. doi: 10.3390/biology11060865 (PMC9220156; doi:10.3390/biology11060865)

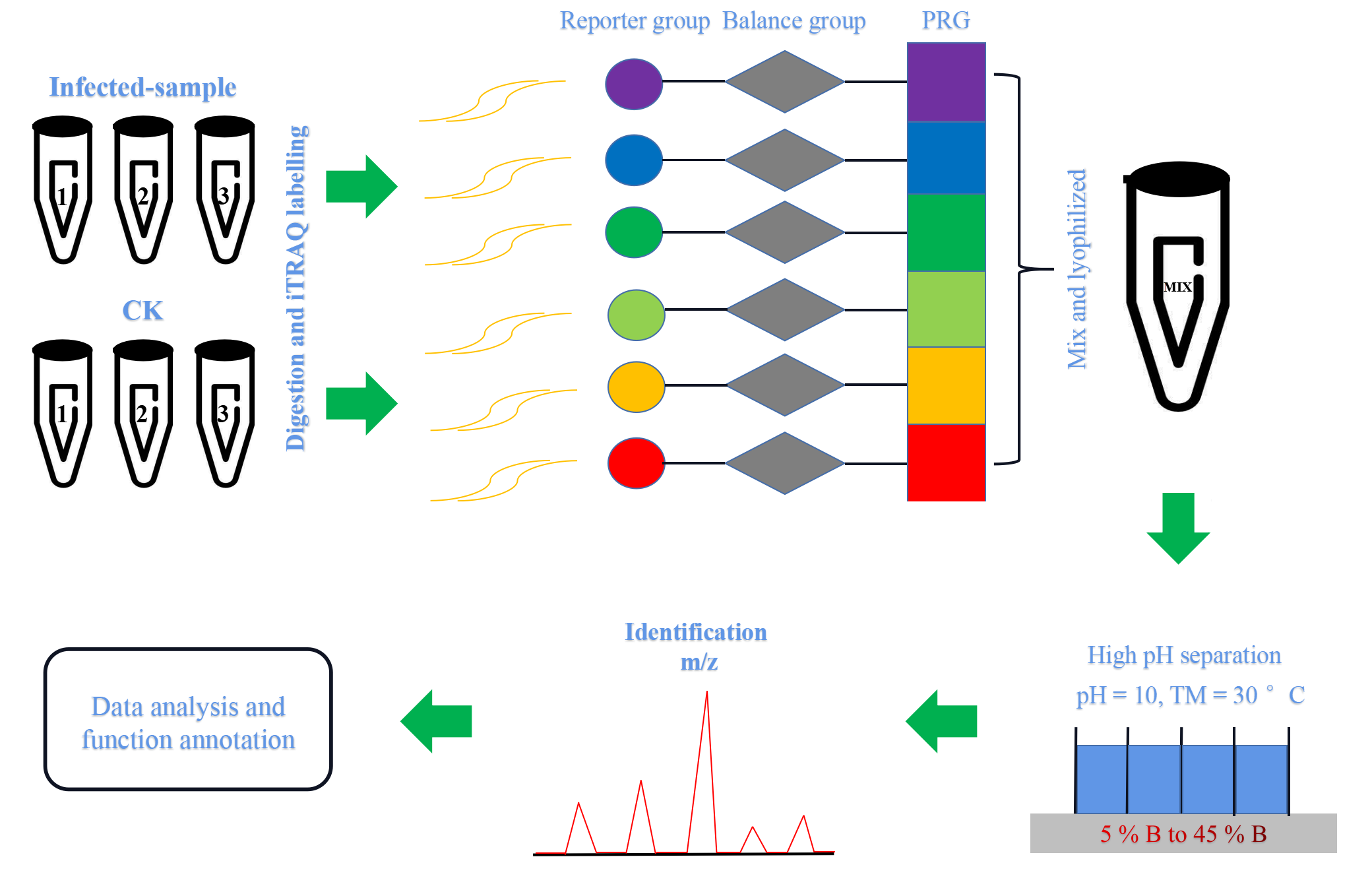

Supplement: Supplementary file 1 [file biology-11-00865-s001.zip › Supplementary Fig S1.tif]

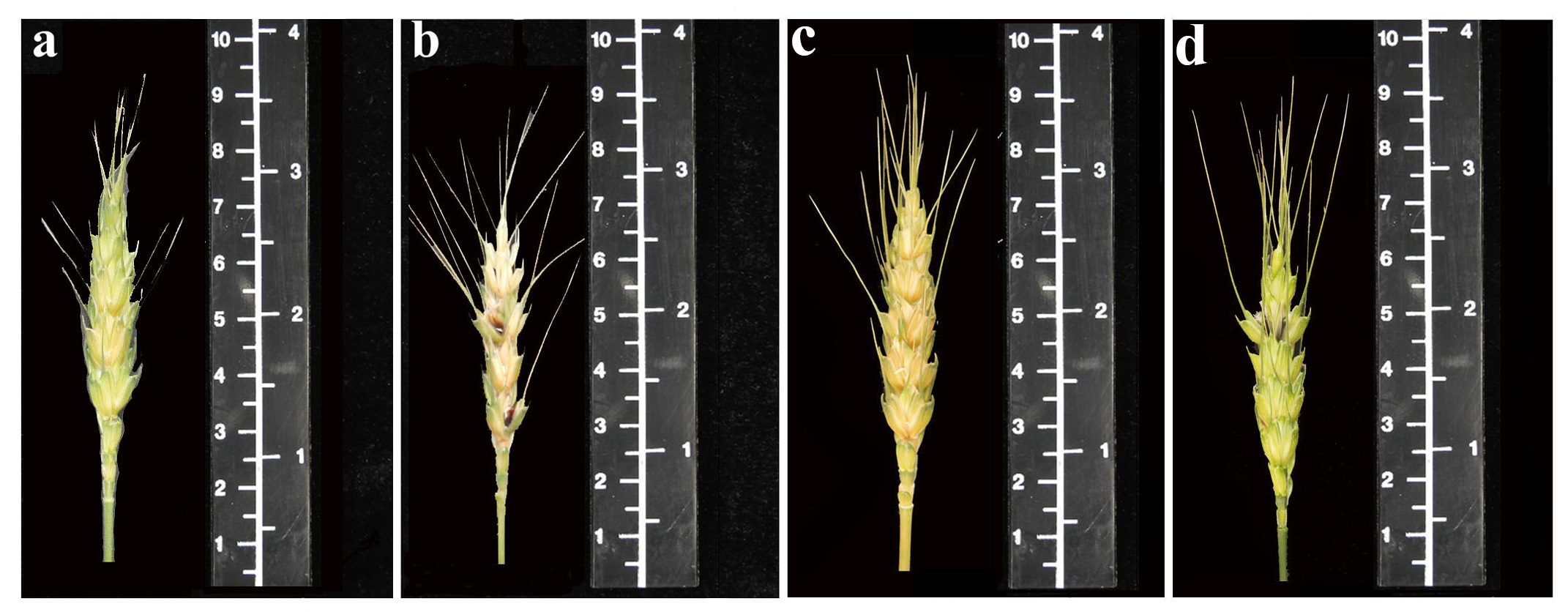

Supplement: Supplementary file 1 [file biology-11-00865-s001.zip › Supplementary Fig S2.tif]
